# Recovery, Molecular Characterization, and Ampelographic Assessment of Marginal Grapevine Germplasm from Southern Umbria (Central Italy)

**DOI:** 10.3390/plants10081539

**Published:** 2021-07-27

**Authors:** Alessandra Zombardo, Paolo Storchi, Paolo Valentini, Alice Ciofini, Daniele Migliaro, Manna Crespan

**Affiliations:** 1 Council for Agricultural Research and Economics, Research Centre for Viticulture and Enology, Viale Santa Margherita 80, 52100 Arezzo, AR, Italy; paolo.storchi@crea.gov.it (P.S.); paolo.valentini@crea.gov.it (P.V.); alice.ciofini@crea.gov.it (A.C.); 2 Council for Agricultural Research and Economics, Research Centre for Viticulture and Enology, Viale 28 Aprile 26, 31015 Conegliano, TV, Italy; daniele.migliaro@crea.gov.it (D.M.); manna.crespan@crea.gov.it (M.C.)

**Keywords:** *Vitis vinifera*, germplasm recovery, genetic identification, SSR markers, ampelography

## Abstract

The protection of grapevine biodiversity and the safeguarding of genetic variability are certainly primary and topical objectives for wine research, especially in territories historically devoted to viticulture. To assess the autochthonous germplasm of three different districts of Southern Umbria (Central Italy), the plant material of 70 grapevines retrieved from reforested land plots or old vineyards was collected, and their genetic identity was investigated using 13 microsatellite markers (SSR). The results revealed the presence of 39 unique genotypes, divided into 24 already-known cultivars and 15 never-reported SSR profiles. Most of the grapevine accessions were then vegetatively propagated and cultivated in a vineyard collection both to be protected from extinction and to be evaluated at the ampelographic level. Overall, this work emphasizes the need for recovering the threatened genetic variability that characterizes minor neglected grapevine cultivars or biotypes of Southern Umbria germplasm, and the requirement to revalue and exploit the more valuable genetic resources to enhance the local agri-food economy.

## 1. Introduction

Umbria is one of the smallest regions in the central part of the Italian Peninsula and is characterized by a landlocked landscape of rolling hills. Because of its pivotal position, Umbria always had important historical roles, often being a territory of disputes between the different neighboring domains, but above all, it was a key hub for major commercial routes and agricultural trades, in both north–south (from and to Rome) and east–west directions (between the Adriatic and Tyrrhenian Seas). Umbria has developed a well-established winemaking tradition that dates to the Etruscan period and persists to this day due to its favorable climate [1]. Until the recent past, wine production in this region was exclusively for family use and was primarily represented by mixed viticulture [2]. Throughout the 1950s, in fact, the most common training system still used was “viti maritate” (or “*arbusta*” according to Plinio il Vecchio), in which the vines are grown on other supporting trees (e.g., poplars) within a promiscuous cropping system [3]. Afterward, starting from the 1960s, there was a decisive change that oriented viticulture towards intensive monoculture and quality winemaking, thanks to the adoption of modern agronomic and oenological techniques [4].

Currently, the wine-growing acreage of Umbria is about 12,000 hectares [5] with two denomination of controlled and guaranteed origin (DOCG) and 13 denomination of controlled origin (DOC) wines, which are rather appreciated on the national and international markets. The main cultivated variety is Sangiovese (20% of the total area), followed by Trebbiano Toscano (12%), Grechetto (11%), Merlot (10%), Sagrantino (7%), and Cabernet Sauvignon (4%). Despite the limited availability of autochthonous cultivars in the official productive panorama, the Umbrian viticulture retains a huge biodiversity heritage [6]. As a matter of fact, several minor grapevine varieties (belonging to known genotypes or showing a unique fingerprinting) can easily be found in abandoned reforested land plots or in old vineyards, and some historical documents describe certain spontaneous vines varieties in wooded areas called “Viti vicciute” [7]. Moreover, there is evidence that several autochthonous varieties are still grown locally, although unnamed and neither identified nor described [8]. The real origins of the different grapevine varieties historically cultivated in Umbria are still unclear, and the presence of sparse wild vines (as *Vitis vinifera* L. subsp. *sylvestris*) is reported [9].

**Figure 1:**
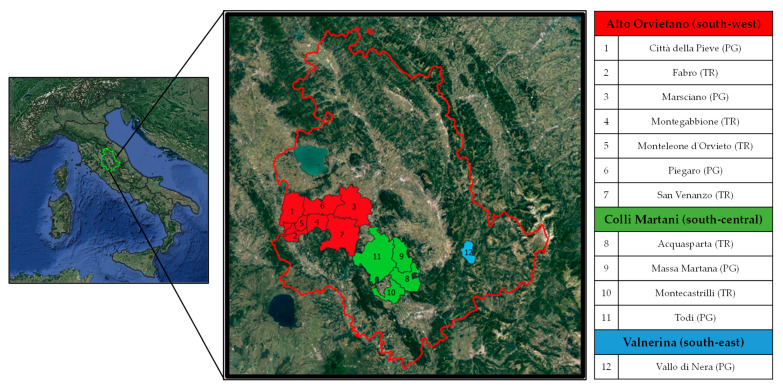
Geographical location and list of the 12 municipalities of Southern Umbria where the 70 grapevine samples included in the study were recovered. The color refers to the three territories considered: Alto Orvietano (in red), Colli Martani (in green), and Valnerina (in light blue). In brackets: abbreviation of the two provinces of Umbria, PG = Perugia; TR = Terni.

In the period 2013–2020, a research project funded by the Umbria Region was developed to protect the traditional germplasm of Umbria. Several inspections were carried out in 12 municipalities included in Alto Orvietano (southwest), Colli Martani (south-central), and Valnerina (southeast) territories of both the provinces of Perugia and Terni in Southern Umbria (Figure 1) to find vines of possible interest in private farms, in old and abandoned vineyards, or in wooded areas no longer devoted to agriculture. This work aimed to search, identify, rescue from genetic erosion, and revive some local grapevine varieties belonging to fragile and disadvantaged agricultural districts that are slowly recovering thanks to the economic boost of food and wine tourism [10].

## 2. Results

### 2.1. Genetic Characterization of Grapevine Germplasm

The molecular analyses were performed on 70 grapevine samples collected in three different districts located in Southern Umbria (Alto Orvietano, Colli Martani, and Valnerina). Thirty-nine genotypes were found (Table 1), with 52 samples belonging to 24 known grapevine varieties and 18 samples showing 15 never-reported SSR profiles (Table 2).

### 2.2. Statistics for SSR Data

Statistics on the analyzed 13 SSRs are shown in Table 3. One hundred and fifteen alleles were detected, with a mean of 8.8 alleles per *locus* and a mean number of effective alleles of 4.9. The mean H_o_ and mean H_e_ were 0.831 and 0.789, respectively.

### 2.3. Genetic Similarity

**Figure 2:**
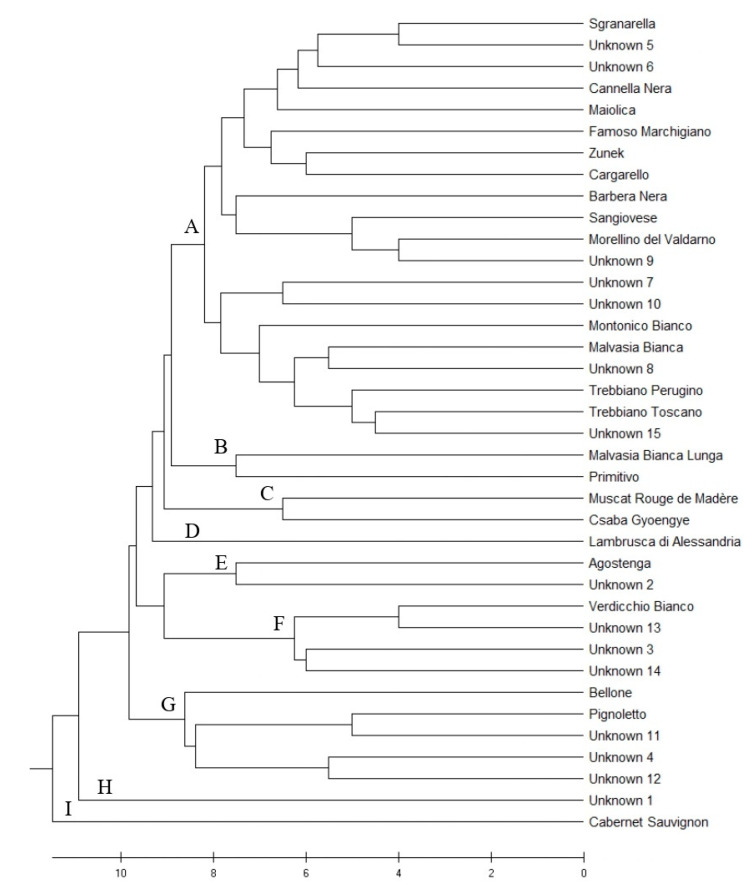
Dendrogram of genetic similarity of 38 genotypes with 12 SSR markers. The optimal tree was inferred using the UPGMA method [15] and MEGA X software [16]. The tree is drawn to scale, with branch lengths in the same units as those of the genetic distances used to infer the tree. The genetic distances were computed using GenAlEx software.

The dendrogram of genetic similarity is shown in Figure 2. Cutting the dendrogram around nine on the X scale, nine groups were obtained. The main group (A) encompassing 20 genotypes (around half of the total genotypes found) includes varieties grown in the nearby Marche Region, such as Sgranarella and Famoso Marchigiano, and predominant Italian varieties such as Sangiovese, Trebbiano Toscano, and Montonico Bianco. Two pairs of varieties are grouped in B, i.e., Malvasia Bianca Lunga and Primitivo, likely of Balkan origin [14]; another two cultivars are in C, i.e., Muscat Rouge de Madère (also called Moscato Violetto) and Csaba Gyoengye (Perla di Csaba), both with muscat flavor and related to Muscat à Petits Grains Blancs as offspring and grandchild, respectively. Lambrusca di Alessandria is the only cultivar in group D. Agostenga groups with Unknown 2 in E. Interestingly, Verdicchio Bianco clusters with three unknown genotypes (Unknown 13, Unknown 3, and Unknown 14) (F); looking at SSR data, the three unknown genotypes share at least one allele per locus with Verdicchio Bianco, therefore they could be parent–offspring (PO) related. Likewise, Pignoletto could be PO-related with Unknown 11 in group G.

Even if collected in a small area, unknown genotypes were shown to be overall highly different and did not group together in the tree. Interestingly, Unknown 1 (H) and Cabernet Sauvignon (I) seem to be outsiders.

### 2.4. Vineyard Collection Establishment and Ampelographic Descriptions

After genetic identification, a first group of 38 samples was vegetatively multiplied by grafting a wood branch on the same rootstock (1103 Paulsen); an experimental vineyard of Southern Umbria germplasm was started in 2015 with the rooted cuttings obtained; in the following years, additional accessions were added to the collection. Currently, the repository hosts 53 grapevine accessions out of the 70 vines considered in this study (Table 1, sample names in bold). In particular, they correspond to 29 different genotypes and, among them, 17 are already known varieties, while 12 are unknown genotypes.

For 38 accessions with already adult vines (over three years old, grape-producing plants), the ampelographic descriptions were performed following the recommended OIV —International Organisation of Vine and Wine methodology, based on the primary descriptor priority list [13]. These 38 accessions (marked with asterisks in Table 1) belong to 20 different genotypes, eight never described before, and 12 already known cultivars. Among the known cultivars, eight are already included in the Italian Catalogue of Grapevine Varieties [12] (i.e., Verdicchio Bianco, Trebbiano Toscano, Sangiovese, Montonico Bianco, Malvasia Bianca Lunga, Cargarello/Canaiolo Bianco, Lambrusca di Alessandria, and Primitivo) and four are not yet enrolled (i.e., Trebbiano Perugino, Famoso Marchigiano, Cannella Nera, and Zunek).

Ampelographic data of the eight unknown genotypes (11 accessions) are presented in Table 4, while those of the 12 already known varieties (27 accessions) are shown in Supplementary file S1. In addition to the 14 characters included in the primary descriptor priority list, we also detected the character OIV 151, related to the sexual organs of the flowers. As reported in Table 4, Casevecchie 2 (Unknown 01) has male flowers with fully developed stamens and reduced gynoecium; consequently, this accession produces very loose bunches with few fully developed berries.

## 3. Discussion

To explore the biodiversity of autochthonous grapevine from Southern Umbria, samples from 70 vines were collected in marginal vineyards not subjected to renewal in the last decades or in wooded areas where they were found as isolated relic plants. Genotyping was applied using a set of 13 SSR markers encompassing the nine SSR markers recommended by the European project GrapeGen06 [17]. The addition of VMC6E1, VMC6F1, VMC6G1 [18], and VMCNG4b9 [19] is a routine methodological approach of our laboratory that increases the resolution of the results and offers additional information to grapevine genotyping [20]. The screening by the internationally recognized microsatellite markers method permitted accurate and univocal identification of 39 different genetic profiles (24 known varieties and 15 unknown genotypes), giving an objective varietal classification [21]. The SSR profiles obtained also allowed the comparison of the Umbrian grapevine germplasm with literature data and molecular databases, searching for synonyms in neighboring countries.

Among the 70 grapevine samples retrieved, Verdicchio Bianco was the most common genotype (nine samples); this variety is rather widespread in Central and Northeastern Italy; in particular, it is one of the most prized white grapes of Marche [22], a region of the Adriatic coast bordering Umbria. Verdicchio Bianco has some officially recognized synonyms [23]: Trebbiano di Soave (appellation mainly used in several provinces of Veneto region), Verdello (the synonym used in Umbria), Verduschia or Duropersico (in Tuscany), and Trebbiano Verde (in Latium). Trebbiano Toscano (or Ugni Blanc) was also commonly recovered, as well as Sangiovese (8 and 7 samples, respectively); they are certainly the major white and red grape cultivar, and historically ubiquitous in the viticultural areas under investigation [24]. Many other recovered genotypes demonstrate the eclectic nature of the viticultural heritage of southern Umbria, which originates from the strategic importance of these territories in the trade routes of the past.

Trebbiano Perugino is a not a very common variety and is often confused with Trebbiano Toscano. The Trebbiano Perugino origin is unknown, but it is counted in ampelographic treatises as early as 1600 and then in the following centuries [25]. In the past, it was mainly present in the province of Perugia. Trebbiano Perugino has a remarkable vigor, and gives abundant and constant grape yields; it is characterized by elongated, large clusters and medium-small golden-yellow berries [25].

Montonico Bianco is an interesting, but now declining, cultivar, used both for wine and as a table grape. It is very resistant to late cold and has medium resistance of leaves and grapes to downy and powdery mildew [26]. Montonico Bianco is grown under a profusion of different names in the Italian Adriatic regions of Abruzzo, Marche, Apulia, but also in Tuscany, Umbria, and Calabria, suggesting a former wide distribution throughout Central and Southern Italy [27,28].

Famoso Marchigiano, different from Famoso from Emilia–Romagna, is a minor variety recently recovered in the Marche region [22]. Sgranarella is a white-berried variety also originating from Marche. It is mentioned in various ampelographic treaties of the past [29] and has recently been enrolled in the Italian Catalogue of Grapevine Varieties (2019) [12]. Both varieties are parent–offspring related with Crepolino/Visparola, one of the main founders of Italian grapevine germplasm [22].

Two “Cornacchione” samples from Valnerina showed an SSR profile different from the Cornacchione reported in the paper of [8] about the ancestral germplasm of Umbria and turned out to correspond to Cannella Nera [30], sometimes listed with the synonym Panfinone, a rare black-berried variety counted in the *V*IVC [11] and lacking any other bibliographic information.

Csaba Gyoengye (also called Perla di Csaba) is an early-ripening white grape variety, not surprisingly called by the keepers “Lugliolo”, namely “grapes that ripen in the month of July”. Csaba Gyoengye, a cross between Madeleine Angevine and Muscat Fleur d’Oranger [31], is widespread, especially in Northern Europe, and is traditionally grown as a table grape for fresh consumption; however, in Hungary, the country of origin, it is also used for winemaking [32]. Some enological trials on Csaba Gyoengye grapes were recently carried out in the territory of Colli Martani, giving interesting perspectives (unpublished data). Thus, based on these results, this cultivar was very recently approved by the Ministry of Agriculture for dual use in Italy, as well [12].

Villard Blanc is a *Vitis* interspecific crossing (Seibel 6468 X Seibel 6905 or Subéreux) of French origin, selected for its resistance to fungal diseases. In fact, it has the *Rpv 3.1* gene in its genome (located in chromosome 18), which confers tolerance to downy mildew [33]. During the last century, direct–producer hybrids were commonly grown, trying to successfully obtain pest tolerance [34], and Villard Blanc was rather widely cultivated in some Italian regions (Veneto, for example). However, since the 1970s, its presence has dropped drastically, and the use of vines with non-*Vitis vinifera* DNA was even banned.

Originally from Slovenia, Zunek is known as Uva Sacra in Italy, where it is produced for table grapes [35]. Within this research, two different accessions from different wine-growing districts of southern Umbria (Alto Orvietano and Valnerina) turned out to be Zunek/Uva Sacra. The relic plants discovered were both capable of producing white grapes, in huge clusters with large and juicy berries, suitable for fresh consumption.

Morellino del Valdarno is a minor Tuscan cultivar native to the province of Arezzo which displayed a first-degree genetic relationship with Sangiovese [36] and showed interesting characteristics in monovarietal enological trials [37]. Since Morellino del Valdarno was also found in the territory under study, it can be considered a variety worth being monitored and safeguarded from the risk of genetic erosion.

Lambrusca di Alessandria is a cultivar native to Piedmont, as deducible from its name: the Alessandria province was indeed the largest growing area [38]. The name (as for Lambrusco) can be traced back to the Latin term *Labruscae* that designates wild grapevines and refers to the typical characteristics of the grapes, with a high content of tannins and anthocyanins. It was recently discovered that Lambrusca di Alessandria had a prominent genetic role as a parent, showing several presumed descendant cultivars [14,39]. Today it is considered as a variety with tiny commercial importance and is widespread exclusively in the oldest vineyards. To our knowledge, its presence in Central Italy is reported here for the first time.

Barbera is another grape variety from Piedmont that we retrieved in Umbria. It is a historical vine, already described in the very first treatises on ampelography; in the 1970s, it was the most common black-berried grape variety in Italy, probably due to its rusticity and ability to tolerate overseas parasites [40]. Currently, the cultivation area is much smaller than in the past and is limited to the suitable areas of Northwest Italy [40]. In the literature, the presence of Barbera and other “new vines introduced from outside that spread in a not indifferent way” is mentioned in the territory of Gubbio (PG), already at the end of the nineteenth century [41]; in Umbria, nowadays, Barbera is used for monovarietal vinification by a few local winegrowers only, for example in Valnerina.

Agostenga is an early-ripening cultivar (as can be guessed from the reference to the month of August in its name) also of Piedmont origin, but it is no longer present there. It is better called Prié Blanc or Blanc de Morgex in Aosta Valley (Northwest Italy), where it is cultivated almost exclusively today [29]. It grows at nearly prohibitive altitudes at the foot of Mont Blanc (900–1200 m above sea level), where there is no phylloxera pressure, therefore it does not require grafting on resistant rootstocks [42]. A few years ago, the synonymy with a geographically distant Spanish cultivar called Legiruela was established, as its SSR profile (34 loci) exhibited a perfect match with Agostenga/Prié Blanc [43].

Other varieties, quite common in Central Italy vineyards, were found: Malvasia Bianca Lunga and Malvasia Bianca, the first more and the second less widespread, respectively; Cargarello or Canaiolo Bianco/Drupeggio, an ancient minor variety part of the viticultural tradition of Tuscany and Umbria [44], currently poorly spread and poorly propagated in nurseries; Pignoletto, so-called in Emilia Romagna but grown as Grechetto Gentile in Umbria; Maiolica, a black-berried ancient cultivar grown in the near Marche region and in Tuscany (where it is given the synonym of Sanforte), offspring of the prolific Cascarello/Visparola and one of the parents of the Apulian Negroamaro [14]; Muscat Rouge de Madère or Moscato Violetto, a pleasant Muscat variety, sporadically present in Tuscany, offspring of Muscat à Petits Grains Blancs and Sciaccarello [14,45]; the Apulian Primitivo, an international black wine variety that probably originated in Montenegro, where it is mainly called Kratošija [46,47], and successfully grown in California as Zinfandel; Bellone, a white variety typical of Latium.

As common in germplasm assessment studies, one prominent “international grape variety” was found, Cabernet Sauvignon, which is grown worldwide thanks to its phenotypic plasticity and adaptability to the environment.

Concerning the 15 unknown genotypes, Rantola 32 accession recovered in San Venanzo (TR) was classified as Unknown 15; this genotype was already found in the province of Arezzo (Tuscany) in 2005 (unpublished data). As for Morellino del Valdarno, in this case we also tracked down, in South Umbria, some grapevines already present in Tuscan winegrowing areas, perhaps widespread vegetatively propagated cultivars in the past, now almost extinct. Casevecchie 2 (Unknown 01) showed the most divergent genotype among those here analyzed (except for Cabernet Sauvignon). The particularity of flowers with fully developed stamens and reduced gynoecium suggests a link with local *Vitis sylvestris* vines.

Alongside the genetic identification of the plant materials found, the propagation and the subsequent cultivation of most of the retrieved vines in a vineyard collection (as a repository) allowed us not only to safeguard several genotypes from extinction and to preserve the already known cultivars as a source of intravarietal diversity, but also to plan the evaluation of the grapes in terms of yields and quality.

## 4. Materials and Methods

### 4.1. Plant Materials

Young leaves of 70 vines were collected in situ in 12 different municipalities in the provinces of Perugia and Terni, covering a total area of about 1100 km^2^ (Figure 1), through various samplings that took place in the time frame of 2013–2019. The vines (mostly seedlings or not-grafted relic plants) were cataloged with a denomination given by local winegrowers or according to the place of discovery (Table 1). In any plant materials recovered, putative variety and grape characteristics (e.g., berry color) were neither already known nor assumed.

### 4.2. SSR Genotyping

Thirteen SSR markers were used for genotyping: the nine proposed as common grape markers for international use within the framework of the Grapegen06 European project (VVS2, VVMD5, VVMD7, VVMD25, VVMD27, VVMD28, VVMD32, VrZAG62, VrZAG79) [17], plus VMC6E1, VMC6F1, VMC6G1 [18], and VMCNG4b9 [19]. The SSR analyses were performed following the protocol detailed in [20]; two internationally required SSR markers (VVMD25 and VVMD32) [17] were also added and the set of 13 SSR markers were analyzed by two multiplex PCRs using fluorescent primers and an ABI3130xl genetic analyzer (Applied Biosystems, Foster City, CA, USA). The presence of PCR products was assessed by electrophoresis in a 1.5% agarose gel and quantified by comparison with a MassRuler DNA ladder mix (Thermo Fisher Scientific, Waltham, MA, USA). PCR products (0.5 μL) were mixed with 9.35 μL of formamide and 0.15 μL of the GeneScan™ 500 LIZ Size Standard (Life Tech, Carlsbad, CA, USA). Capillary electrophoresis was conducted in an ABI 3130xl Genetic Analyzer (Life Tech, CA, USA). Allele calling was performed with GeneMapper 5.0, using the 500 LIZ size standard as an internal ladder and a homemade bin set built with reference varieties. Allele sizes were recorded in bp (using the *V*IVC allele sizing [11]), and genotypes showing a single peak at a given locus were considered homozygous.

Identifications were performed by comparing the obtained SSR profiles with the CREA Viticulture and Enology molecular database (which currently contains about 5000 unique profiles, and is constantly updated), literature information, and the *Vitis* International Variety Catalogue [11]. In detail, Famoso Marchigiano, Morellino del Valdarno, Drupeggio, and Cannella Nera were identified referring to [22,30,36,44], respectively; Zunek, Csaba Gyongye, Villard Blanc, and Muscat Rouge de Madère were identified through the *V*IVC [11]; Trebbiano Perugino using the CREA-VE molecular database; all the remaining varieties using the molecular database of the Italian Catalogue of Grapevine Varieties [12].

### 4.3. Statistics on SSR Markers

Cervus 3.0 software [48] and GenAlEx 6.5 software [49] were used to determine the number of different alleles (N_o_ alleles), effective number of alleles (N_e_ alleles), observed (H_o_), expected heterozygosity (H_e_), polymorphic information content (PIC), Hardy–Weinberg equilibrium (HW), probability of null alleles (F (null)), and identity probability between two unrelated individuals and between two hypothetical full siblings (P_ID_ and P_ID_SIBS).

### 4.4. Dendrogram of Genetic Similarities

Genetic distances were computed using GenAlEx 6.5 software [49,50] and a dendrogram of genetic similarity was computed using 38 genotypes (Villard Blanc was excluded for being a hybrid) and 12 SSR markers (VMC6F1 was excluded due to missing data). The optimal tree was inferred using the UPGMA method [15] and MEGA X software [16].

### 4.5. Grapevine Germplasm Collection

An experimental germplasm collection of 2000 m^2^ was set up from 2015 until 2020 at Castello di Montegiove Estate in the municipality of Montegabbione (province of Terni, Umbria, Italy; 44°91′80″ N, 12°15′66″ E). The vines, grafted on 1103 Paulsen rootstock, were planted with an east–west orientation; planting distances were 0.90 m within rows and 2.80 m between rows. The vines were trained on upward vertical shoot positioned trellis, with spur cordon pruning and an average of 10 buds per vine. Over the years, the vineyard was conducted with homogeneous agronomic conditions, and pest management was scheduled with calendar sprays at 10-day intervals. For each of the 53 accessions actually present (sample names in bold in Table 1), there are at least 30 vines; Sangiovese and Trebbiano Toscano were added as reference cultivars for Central Italy grapevine germplasm.

### 4.6. Ampelographic Description

The ampelographic description of the main grapevine morphological traits was carried out in the experimental vineyard in Montegabbione (TR, Italy) during the vegetative season of 2019 on adult vines (over 3 years old), according to the set of 14 standardized OIV primary descriptor priority list [13]: young shoot (OIV 001, 004), shoot (OIV 016), young leaf (OIV 051), mature leaf (OIV 067, 068, 070, 076, 079, 081-2, 084, 087), berry (OIV 223, 225); as reputed interesting, the OIV 151 characteristic about flower sexual organs was added to the list.

## 5. Conclusions

The present study made it possible to explore a small part of the grapevine biodiversity of Southern Umbria by highlighting the hypothetical composition of the autochthonous germplasm that survived the phylloxera epidemics of the late 1800s and the widespread diffusion of the most common international varieties from the second half of the 20th century.

The 15 unknown genotypes recovered thanks to the present research work will soon be officially added to the VIVC [11], and the evaluations of their ampelographic, agronomic, and oenological characteristics are underway. Once enough data is collected, we plan to compare the available literature information about the old grapevine germplasm of Umbria to attribute the potential identity to any vines historically present in the districts under investigation.

The rediscovery and preservation of indigenous endangered grapevine cultivars or biotypes, especially in marginal areas such as the districts of Southern Umbria considered (Alto Orvietano, Colli Martani, and Valnerina), can give benefits to the local wine industry providing the basis for niche wines, an added value that can definitely help to relaunch the local economy in the territory of origin.

## Figures and Tables

**Table 1:** List of the 70 grapevine samples grouped by their genotype. Original locations (municipality and province), berry color, true-to-type prime name, SSR profile ID number, and *Vitis* International Variety Catalogue [11] code are reported. In brackets: Italian common synonyms of some grapevine cultivars, according to the Italian Catalogue of Grapevine Varieties [12].

Sample Name	Original Location: Municipality (Province)	Berry Color	True-to-Type Prime Name	SSR Profile ID	*V*IVC Code
Known Grapevine Cultivars					
Cerasuolo	Montegabbione (TR)	white	Verdicchio Bianco	1	12963
Fontana *	Montegabbione (TR)				
Montarale Testa 2 *	Piegaro (PG)				
Montarale	Piegaro (PG)				
Pisano 28 *	Fabro (TR)				
**Scarzuola 6**	Montegabbione (TR)				
**Scarzuola 7**	Montegabbione (TR)				
Terracavata 24	Montegabbione (TR)				
Palornie	Montecastrilli (TR)				
Cantagallina 1 *	San Venanzo (TR)	white	Trebbiano Toscano	2	12628
Cantagallina 3 *	San Venanzo (TR)				
**Cerqueto 2**	Marsciano (PG)				
**Cerqueto 3**	Marsciano (PG)				
Pobeto 1 *	Montegabbione (TR)				
Pomario	Piegaro (PG)				
Rantola 31 *	San Venanzo (TR)				
Terracavata 23 *	Montegabbione (TR)				
Casevecchie 1 *	Montegabbione (TR)	black	Sangiovese	3	10680
Fontesecca 1 *	Città della Pieve (PG)				
Fontesecca 2 *	Città della Pieve (PG)				
**Scarzuola 2**	Montegabbione (TR)				
Scarzuola 3	Montegabbione (TR)				
Scatolla 21 *	Montegabbione (TR)				
Siliano *	Città della Pieve (PG)				
Fontesecca 3 *	Città della Pieve (PG)	white	Trebbiano Perugino	4	12624
San Lorenzo Fontani *	Monteleone d’Orvieto (TR)				
Cantagallina 2 *	San Venanzo (TR)	white	Famoso Marchigiano	5	-
Montarale Testa 1 *	Piegaro (PG)				
Pizziconi 1 *	Fabro (TR)	white	Montonico Bianco	6	7960
Rantola 30 *	San Venanzo (TR)				
Cornacchione 1 *	Vallo di Nera (PG)	black	Cannella Nera	7	-
Cornacchione 2 *	Vallo di Nera (PG)				
Pizzutello bianco *	Vallo di Nera (PG)	white	Zunek	8	17739
**Montegiove**	Montegabbione (TR)				
Montarale Vergari 1 *	Piegaro (PG)	black	Lambrusca di Alessandria	9	6688
Montarale Vergari 2 *	Piegaro (PG)				
**Pizziconi 2**	Fabro (TR)	black	Cabernet Sauvignon	10	1929
Palombaro Avi Amonzi *	Piegaro (TR)	white	Malvasia Bianca Lunga	11	7262
**Monteleone**	Monteleone d’Orvieto (TR)	white	Malvasia Bianca	12	7256
Scarzuola 1	Montegabbione (TR)	black	Muscat Rouge de Madère	13	8249
Scarzuola 4	Montegabbione (TR)	black	Barbera Nera	14	974
**Cerqueto 1**	Marsciano (PG)	white	Pignoletto	15	9254
Terracavata 25 *	Montegabbione (TR)	white	Cargarello (Canaiolo Bianco or Drupeggio)	16	2092
**Scarzuola 5**	Montegabbione (TR)	white	Villard Blanc	17	13081
Pobeto 2	Montegabbione (TR)	black	Morellino del Valdarno	18	8457
Casevecchie 3 *	Montegabbione (TR)	black	Primitivo	19	9703
Uva Palazza	Todi (PG)	white	Bellone	20	1114
Lugliolo Manni	Todi (PG)	white	Agostenga (Prié Blanc)	21	107
Lugliolo Leonucci	Massa Martana (TR)	white	Csaba Gyoengye (Perla di Csaba)	22	9166
Bacarello	Acquasparta (TR)	white	Sgranarella	23	26656
Rosaro	Acquasparta (TR)	black	Maiolica	24	7136
Unknown Grapevine Cultivars					
Casevecchie 2 *	Montegabbione (TR)	black	Unknown 01	25	-
**Terracavata 22**	Montegabbione (TR)	nyd	Unknown 02	26	-
Pian del sette *	Montegabbione (TR)	black	Unknown 03	27	-
Pornellese *	San Venanzo (TR)	black	Unknown 04	28	-
Rantola 34 *	San Venanzo (TR)	white	Unknown 05	29	-
Scatolla 20 *	Montegabbione (TR)				
Verdone 1 *	Vallo di Nera (PG)	white	Unknown 06	30	-
Verdone 2 *	Vallo di Nera (PG)				
Uva genia 1 *	Vallo di Nera (PG)	white	Unknown 07	31	-
Uva genia 2 *	Vallo di Nera (PG)				
Cerqueto 4	Marsciano (PG)	nyd	Unknown 08	32	-
**Pianello**	Montegabbione (TR)	black	Unknown 09	33	-
Castel di Fiori *	Montegabbione (TR)	white	Unknown 10	34	-
**Francescame**	Montegabbione (TR)	nyd	Unknown 11	35	-
**Pisano 33**	Fabro (TR)	black	Unknown 12	36	-
**Uva propria**	Todi (PG)	white	Unknown 13	37	-
Sciuttarella	Todi (PG)				
Montenero	Todi (PG)	nyd	Unknown 14	38	-
Rantola 32 *	San Venanzo (TR)	black	Unknown 15	39	-

The sample names in bold correspond to the 53 vine accessions currently present in the experimental vineyard. The asterisks (*) indicate 38 accessions with already adult vines (over 3 years old) that were subjected to ampelographic description according to OIV primary descriptor priority list [13]. Nyd = not yet determined berry color.

**Table 2:** List of 39 unique genetic profiles obtained at 13 SSR loci.

SSR Profile ID	Grapevine Variety	VVS2		VVMD5		VVMD7		VVMD25		VVMD27		VVMD28		VVMD32		VrZAG62		VrZAG79		VMC6E1		VMC6F1		VMC6G1		VMCNG4b9	
1	Verdicchio Bianco	133	155	230	242	239	247	241	241	180	186	236	258	252	256	196	196	249	257	165	165	135	139	169	197	164	166
2	Trebbiano Toscano	133	143	228	234	249	253	241	255	180	184	244	248	250	272	194	200	245	251	141	161	133	139	177	187	162	176
3	Sangiovese	133	133	228	238	239	263	241	241	180	186	234	244	252	256	194	196	243	259	143	165	139	139	177	197	158	168
4	Trebbiano Perugino	133	155	228	238	249	253	241	255	180	192	236	258	250	252	200	200	247	251	161	165	133	139	177	187	158	176
5	Famoso Marchigiano	133	155	228	230	239	249	241	255	192	195	244	248	256	262	196	200	247	249	141	161	133	139	177	197	150	158
6	Montonico Bianco	143	145	234	234	239	249	241	255	180	186	246	248	250	252	188	200	251	251	141	169	139	145	169	177	158	176
7	Cannella Nera	133	133	228	228	239	247	239	241	184	186	236	248	272	272	188	204	247	251	143	157	133	139	169	197	150	158
8	Zunek	133	137	228	242	239	247	241	241	180	184	248	258	262	272	190	196	243	247	165	169	139	145	177	187	150	162
9	Lambrusca di Alessandria	143	151	228	242	253	255	249	255	180	195	228	268	264	272	200	204	251	251	141	157	131	139	177	197	152	172
10	Cabernet Sauvignon	139	151	234	242	239	239	239	249	176	190	234	236	240	240	188	194	247	247	141	165	133	139	169	191	168	176
11	Malvasia Bianca Lunga	145	145	228	242	239	253	239	241	180	180	248	254	252	256	196	200	243	251	143	165	133	139	177	177	150	176
12	Malvasia Bianca	133	133	228	228	239	253	241	255	180	184	236	236	258	272	194	200	245	251	165	169	133	139	169	177	158	162
13	Muscat Rouge de Madère	133	133	228	230	247	249	241	249	180	184	246	258	252	272	186	204	245	255	141	161	133	133	169	177	158	158
14	Barbera Nera	133	135	228	228	249	253	239	255	186	190	234	260	252	272	192	200	243	259	143	151	133	133	169	177	158	172
15	Pignoletto	133	145	228	248	249	263	241	241	186	190	236	258	252	258	200	202	243	251	151	165	133	139	187	187	150	168
16	Cargarello	133	145	230	242	239	249	239	241	182	184	244	258	252	272	188	190	247	259	161	165	139	139	187	197	150	158
17	Villard Blanc	133	143	234	238	237	251	241	255	182	190	234	236	240	256	179	194	255	261	133	143	139	139	187	197	150	158
18	Morellino del Valdarno	133	143	228	238	239	247	241	255	186	190	244	244	240	256	196	204	243	245	143	165	133	139	169	197	168	176
19	Primitivo	133	143	228	238	247	249	239	239	180	182	248	258	256	264	200	204	237	259	141	165	139	139	177	177	150	165
20	Bellone	135	145	230	234	239	247	239	241	180	180	234	260	252	258	188	204	251	259	143	169	139	145	187	187	168	176
21	Agostenga	133	155	230	240	233	247	239	249	186	190	234	244	252	272	194	196	239	251	141	151	133	133	169	169	150	158
22	Csaba Gyoengye	133	155	238	238	247	249	241	241	180	182	218	268	272	272	186	204	255	259	161	165	133	139	169	197	150	158
23	Sgranarella	133	133	228	248	239	249	241	241	195	195	236	244	250	272	196	200	249	251	141	143	133	139	187	197	150	176
24	Maiolica	133	151	228	238	239	249	241	263	180	186	236	236	252	272	196	202	251	259	141	143	131	139	177	187	150	168
25	Unknown 01	133	155	234	242	247	247	241	255	184	192	236	236	240	250	204	204	245	251	141	151	131	147	177	177	162	162
26	Unknown 02	133	151	230	230	257	263	241	255	192	195	234	244	240	240	196	202	251	251	151	159	133	133	169	169	158	162
27	Unknown 03	151	155	230	230	239	249	241	255	180	192	234	258	250	256	196	200	251	257	159	165	133	135	197	197	164	166
28	Unknown 04	133	135	238	240	239	261	241	255	180	190	228	234	252	256	196	204	251	259	145	165	133	139	177	187	158	158
29	Unknown 05	133	133	228	248	239	249	239	241	186	195	244	248	262	272	188	194	249	251	143	143	133	139	187	197	150	158
30	Unknown 06	133	145	228	234	239	243	241	241	186	190	236	244	256	272	188	196	249	251	141	151	133	139	187	197	166	176
31	Unknown 07	143	151	228	234	249	257	249	255	190	195	244	248	258	262	194	200	251	251	143	165	133	139	177	177	150	176
32	Unknown 08	133	133	228	228	239	253	241	241	180	186	244	258	256	272	188	200	251	251	141	169	Md	Md	177	177	158	176
33	Unknown 09	133	143	228	242	239	247	241	255	180	190	234	236	256	272	196	204	243	259	143	165	139	145	169	177	168	176
34	Unknown 10	133	143	228	242	249	249	241	255	195	195	234	244	250	262	194	196	251	251	143	165	133	145	169	187	158	168
35	Unknown 11	145	155	234	248	247	263	241	255	190	190	234	236	240	252	200	202	251	251	159	165	Md	Md	187	187	150	162
36	Unknown 12	135	141	230	240	261	263	249	255	190	190	228	236	240	252	194	204	251	251	145	159	Md	Md	187	187	158	158
37	Unknown 13	133	143	230	242	239	247	241	255	186	186	248	258	256	272	196	196	249	259	151	165	Md	Md	169	197	150	166
38	Unknown 14	133	155	230	230	247	247	241	267	180	184	236	258	252	262	196	204	251	257	159	165	Md	Md	169	197	158	166
39	Unknown 15	133	143	234	238	239	253	241	255	184	195	236	244	250	252	194	200	245	251	143	165	139	151	177	187	158	178

Allele lengths are expressed in base pairs. Allele lengths for VVS2, VVMD5, VVMD7, VVMD25, VVMD27, VVMD28, VVMD32, VrZAG62, and VrZAG79 are provided using the *V*IVC allele sizing. Md = missing data.

**Table 3:** Statistics on the 13 SSR markers analyzed.

Locus	LG	No. of Obs	N_o_ Alleles	N_e_ Alleles	H_o_	H_e_	PIC	HW	F(Null)	P_ID_	P_ID_ SIBS
VVS2	11	39	9	4	0.795	0.732	0.696	NS	−0.0565	1.0 × 10^−1^	4.1 × 10^−1^
VVMD5	16	39	7	5	0.769	0.81	0.774	ND	0.0177	7.4 × 10^−2^	3.8 × 10^−1^
VVMD7	7	39	12	5	0.897	0.817	0.781	ND	−0.0583	5.9 × 10^−2^	3.6 × 10^−1^
VVMD25	11	39	6	3	0.769	0.657	0.599	NS	−0.0935	1.9 × 10^−1^	4.8 × 10^−1^
VVMD27	5	39	8	6	0.821	0.837	0.804	ND	0.0014	5.0 × 10^−2^	3.5 × 10^−1^
VVMD28	3	39	11	6	0.897	0.851	0.820	ND	−0.0316	4.7 × 10^−2^	3.4 × 10^−1^
VVMD32	4	39	8	6	0.897	0.843	0.811	ND	−0.0408	4.6 × 10^−2^	3.4 × 10^−1^
VrZAG62	7	39	10	6	0.897	0.841	0.809	ND	−0.0388	5.1 × 10^−2^	3.5 × 10^−1^
VrZAG79	5	39	11	4	0.769	0.783	0.754	NS	0.0098	7.3 × 10^−2^	3.8 × 10^−1^
VMC6E1	14	39	10	6	0.949	0.843	0.812	ND	−0.0694	4.8 × 10^−2^	3.5 × 10^−1^
VMC6F1	2	34	7	3	0.765	0.658	0.586	NS	−0.095	1.9 × 10^−1^	4.7 × 10^−1^
VMC6G1	11	39	5	4	0.692	0.76	0.705	ND	0.0421	1.1 × 10^−1^	4.1 × 10^−1^
VMCNG4b9	6	39	11	6	0.897	0.833	0.802	ND	−0.0452	5.0 × 10^−2^	3.5 × 10^−1^
Total			115	64						1.6 × 10^−15^	3.4 × 10^−6^
Mean values			8.8	4.9	0.831	0.789	0.750				

LG = linkage group; No. of obs = number of genotypes analyzed to calculate the statistics; No alleles = number of different alleles; Ne alleles = effective number of alleles; Ho, He = observed and expected heterozygosity; PIC = polymorphic information content; HW = Hardy–Weinberg equilibrium: NS = not significant, ND = not done; F (Null) = probability of null alleles; PID and PIDSIBS = identity probability between two unrelated individuals and between two hypothetical full siblings.

**Table 4:** Ampelographic descriptions of 11 grapevine accessions belonging to eight different unknown genotypes present in the vineyard germplasm collection at Castello di Montegiove Estate, municipality of Montegabbione (TR). The set of 14 standardized OIV primary descriptor (PDs) plus OIV 151, grouped according to each target vine organ (young shoot: OIV 001, 004; shoot: OIV 016; young leaf: OIV 051; mature leaf: OIV 067, 068, 070, 076, 079, 081-2, 084, 087; berry: OIV 223, 225; flower: OIV 151), are shown for each SSR profile.

Sample Name			Casevecchie 2	Pian del Sette	Pornellese	Rantola 34 Scatolla 20	Verdone 1 Verdone 2	Uva genia 1 Uva genia 2	Castel di Fiori	Rantola 32
SSR Profile ID			26 Unknown 01	28 Unknown 03	29 Unknown 04	30 Unknown 05	31 Unknown 06	32 Unknown 07	35 Unknown 10	39 Unknown 15
PDs	OIV Code	Characteristic	Note; Description	Note; Description	Note; Description	Note; Description	Note; Description	Note; Description	Note; Description	Note; Description
Young shoot	1	Opening of the shoot tip	5; fully open	5; fully open	5; fully open	5; fully open	5; fully open	5; fully open	5; fully open	5; fully open
	4	Density of prostrate hairs on the shoot tip	5; medium	5; medium	5; medium	3; low	3; low	4; between low and medium	5; medium	7; high
Shoot	16	Number of consecutive tendrils	1; 2 or less	1; 2 or less	1; 2 or less	1; 2 or less	1; 2 or less	1; 2 or less	1; 2 or less	1; 2 or less
Young leaf	51	Color of upper side of blade (4th leaf)	3; bronze	3; bronze	1; green	1; green	2; yellow	3; bronze	1; green	1; green
Mature leaf	67	Shape of blade	2; wedge-shaped	3; pentagonal	2; wedge-shaped	2; wedge-shaped	3–4; pentagonal-circular	3; pentagonal	4; circular	3; pentagonal
	68	Number of lobes	3; five	3; five	3; five	3; five	4; seven	3; five	3; five	3; five
	70	Area of anthocyanin coloration of main veins on upper side of blade	1; absent	3; up to the 1st bifurcation	1; absent	1; absent	1; absent	3; up to the 1st bifurcation	1; absent	1; absent
	76	Shape of teeth	5; mixture between both sides straight and both sides convex	3; both sides convex	5; mixture between both sides straight and both sides convex	3; both sides convex	5; mixture between both sides straight and both sides convex	5; mixture between both sides straight and both sides convex	5; mixture between both sides straight and both sides convex	3; both sides convex
	79	Degree of opening/overlapping of petiole sinus	3; open	5; closed	3; open	3; open	3; open	3; open	5; closed	5; closed
	081-2	Petiole sinus base limited by vein	1; not limited	2; on one side	1; not limited	2; on one side	1; not limited	1; not limited	1; not limited	1; not limited
	84	Density of prostrate hairs between main veins on lower side of blade	5; medium	3; low	3; low	1; none or very low	3; low	3; low	3; low	5; medium
	87	Density of erect hairs on main veins on lower side of blade	1; none or very low	1; none or very low	1; none or very low	1; none or very low	1; none or very low	5; medium	1; none or very low	5; medium
Berry	223	Shape	2; globose	2; globose	3; broad ellipsoid	2; globose	2; globose	2; globose	2; globose	2; globose
	225	Color of skin	6; blue black	6; blue black	6; blue black	1; green yellow	1; green yellow	1; green yellow	1; green yellow	6; blue black
Flower	151	Sexual organs	2; fully developed stamens and reduced gynoecium	3; fully developed stamens and fully developed gynoecium	3; fully developed stamens and fully developed gynoecium	3; fully developed stamens and fully developed gynoecium	3; fully developed stamens and fully developed gynoecium	3; fully developed stamens and fully developed gynoecium	3; fully developed stamens and fully developed gynoecium	3; fully developed stamens and fully developed gynoecium

## Data Availability

The original data presented in this research work are stored in the databases of CREA—Research Centre for Viticulture and Enology.

## Supplementary Materials

The following are available online at https://www.mdpi.com/article/10.3390/plants10081539/s1, Supplementary file S1: Ampelographic descriptions of 27 grapevine samples belonging to twelve different known genotypes present in the vineyard germplasm collection at Castello di Montegiove Estate, municipality of Montegabbione (TR, Italy). Sangiovese and Trebbiano Toscano were added as reference cultivars. The tables contain the set of 14 standardized OIV primary descriptors plus OIV 151, grouped according to each target vine organ (young shoot: OIV 001, 004; shoot: OIV 016; young leaf: OIV 051; mature leaf: OIV 067, 068, 070, 076, 079, 081-2, 084, 087; berry: OIV 223, 225, flower: OIV 151) for each SSR profile.
