# Supplementary material for: Recovery, Molecular Characterization, and Ampelographic Assessment of Marginal Grapevine Germplasm from Southern Umbria (Central Italy)
Source: Plants (Basel). 2021 Jul 27;10(8):1539. doi: 10.3390/plants10081539 (PMC8398187; doi:10.3390/plants10081539)
Supplement: Supplementary file 1 [file plants-10-01539-s001.zip › plants-1296884-supplementary.pdf]

Supplementary file S1. Ampelographic descriptions of 27 grapevine samples belonging to twelve different known genotypes present in the vineyard germplasm collection at Castello di Montegiove Estate, municipality of Montegabbione (TR, Italy). Sangiovese and Trebbiano Toscano were added as reference cultivars. The tables contain the set of 14 standardized OIV primary descriptors plus OIV 151, grouped according to each target vine organ (young shoot: OIV 001, 004; shoot: OIV 016; young leaf: OIV 051; mature leaf: OIV 067, 068, 070, 076, 079, 081-2, 084, 087; berry: OIV 223, 225, flower: OIV 151) for each SSR profile.

Sample name: Montarale Testa 2; Fontana; Pisano 28.
True-to-type prime name: Verdicchio Bianco
SSR profile ID: 1

	OIV Code	Characteristic	Note	Description
Young shoot	001	Opening of the shoot tip	5	fully open
	004	Density of prostrate hairs on the shoot tip	5	medium
Shoot	016	Number of consecutive tendrils	1	2 or less
Young leaf	051	Color of upper side of blade (4th leaf)	1	green
Mature leaf	067	Shape of blade	3-5	pentagonal - circular
	068	Number of lobes	2-3	three - five
	070	Area of anthocyanin coloration of main veins on upper side of blade	1	absent
	076	Shape of teeth	2	both sides straight
	079	Degree of opening/overlapping of petiole sinus	5-7	closed - overlapped
	081-2	Petiole sinus base limited by vein	1	not limited
	084	Density of prostrate hairs between main veins on lower side of blade	5	medium
	087	Density of erect hairs on main veins on lower side of blade	1	none or very low
Berry	223	Shape	2	globose
	225	Color of skin	1	green yellow
Flower	151	Sexual organs	3	fully developed stamens and fully developed gynoecium

Sample name: Cantagallina 1; Cantagallina 3; Pobeto 1; Rantola 31; Terracavata 23; Trebbiano Toscano (reference cultivar).
True-to-type prime name: Trebbiano Toscano
SSR profile ID: 2

	OIV Code	Characteristic	Note	Description
Young shoot	001	Opening of the shoot tip	5	fully open
	004	Density of prostrate hairs on the shoot tip	5	medium
Shoot	016	Number of consecutive tendrils	1	2 or less
Young leaf	051	Color of upper side of blade (4th leaf)	1-2	green - yellow
Mature leaf	067	Shape of blade	3	pentagonal
	068	Number of lobes	2-3	three - five
	070	Area of anthocyanin coloration of main veins on upper side of blade	1	absent
	076	Shape of teeth	5	mixture between both sides straight (note 2) and both sides convex (note 3)
	079	Degree of opening/overlapping of petiole sinus	3	open
	081-2	Petiole sinus base limited by vein	1	not limited
	084	Density of prostrate hairs between main veins on lower side of blade	5	medium
	087	Density of erect hairs on main veins on lower side of blade	1	none or very low
Berry	223	Shape	2	globose
	225	Color of skin	1	green yellow
Flower	151	Sexual organs	3	fully developed stamens and fully developed gynoecium

Sample name: Casevecchie 1; Fontesecca 1; Fontesecca 2; Scatolla 21; Siliano; Sangiovese (reference cultivar).
True-to-type prime name: Sangiovese
SSR profile ID: 3

	OIV Code	Characteristic	Note	Description
Young shoot	001	Opening of the shoot tip	5	fully open
	004	Density of prostrate hairs on the shoot tip	3	low
Shoot	016	Number of consecutive tendrils	1	2 or less
Young leaf	051	Color of upper side of blade (4th leaf)	1	green
Mature leaf	067	Shape of blade	3	pentagonal
	068	Number of lobes	2-3	three - five
	070	Area of anthocyanin coloration of main veins on upper side of blade	1	absent
	076	Shape of teeth	2	both sides straight
	079	Degree of opening/overlapping of petiole sinus	3	open
	081-2	Petiole sinus base limited by vein	1	not limited
	084	Density of prostrate hairs between main veins on lower side of blade	1	none or very low
	087	Density of erect hairs on main veins on lower side of blade	1	none or very low
Berry	223	Shape	2	globose
	225	Color of skin	6	blue black
Flower	151	Sexual organs	3	fully developed stamens and fully developed gynoecium

Sample name: Fontesecca 3; San Lorenzo Fontani.
True-to-type prime name: Trebbiano Perugino
SSR profile ID: 4

	OIV Code	Characteristic	Note	Description
Young shoot	001	Opening of the shoot tip	5	fully open
	004	Density of prostrate hairs on the shoot tip	5	medium
Shoot	016	Number of consecutive tendrils	1	2 or less
Young leaf	051	Color of upper side of blade (4th leaf)	1	green
Mature leaf	067	Shape of blade	3	pentagonal
	068	Number of lobes	3	five
	070	Area of anthocyanin coloration of main veins on upper side of blade	1	absent
	076	Shape of teeth	2	both sides straight
	079	Degree of opening/overlapping of petiole sinus	5	closed
	081-2	Petiole sinus base limited by vein	1	not limited
	084	Density of prostrate hairs between main veins on lower side of blade	7	high
	087	Density of erect hairs on main veins on lower side of blade	5	medium
Berry	223	Shape	2	globose
	225	Color of skin	1	green yellow
Flower	151	Sexual organs	3	fully developed stamens and fully developed gynoecium

Sample name: Cantagallina 2; Montarale Testa 1.
True-to-type prime name: Famoso Marchigiano
SSR profile ID: 5

	OIV Code	Characteristic	Note	Description
Young shoot	001	Opening of the shoot tip	5	fully open
	004	Density of prostrate hairs on the shoot tip	5	medium
Shoot	016	Number of consecutive tendrils	1	2 or less
Young leaf	051	Color of upper side of blade (4th leaf)	1	green
Mature leaf	067	Shape of blade	3	pentagonal
	068	Number of lobes	3	five
	070	Area of anthocyanin coloration of main veins on upper side of blade	1	absent
	076	Shape of teeth	3	both sides convex
	079	Degree of opening/overlapping of petiole sinus	3	open
	081-2	Petiole sinus base limited by vein	3	on both sides
	084	Density of prostrate hairs between main veins on lower side of blade	5	medium
	087	Density of erect hairs on main veins on lower side of blade	3	low
Berry	223	Shape	2	globose
	225	Color of skin	1	green yellow
Flower	151	Sexual organs	3	fully developed stamens and fully developed gynoecium

Accession name: Pizziconi 1; Rantola 30.
True-to-type prime name: Montonico Bianco
SSR profile ID: 6

	OIV Code	Characteristic	Note	Description
Young shoot	001	Opening of the shoot tip	5	fully open
	004	Density of prostrate hairs on the shoot tip	5	medium
Shoot	016	Number of consecutive tendrils	1	2 or less
Young leaf	051	Color of upper side of blade (4th leaf)	3	bronze
Mature leaf	067	Shape of blade	3-4	pentagonal - circular
	068	Number of lobes	3	five
	070	Area of anthocyanin coloration of main veins on upper side of blade	2	only at the petiolar point
	076	Shape of teeth	2	both sides straight
	079	Degree of opening/overlapping of petiole sinus	5	closed
	081-2	Petiole sinus base limited by vein	1	not limited
	084	Density of prostrate hairs between main veins on lower side of blade	3	low
	087	Density of erect hairs on main veins on lower side of blade	3	low
Berry	223	Shape	2	globose
	225	Color of skin	1	green yellow
Flower	151	Sexual organs	3	fully developed stamens and fully developed gynoecium

Sample name: Cornacchione 1; Cornacchione 2.
True-to-type prime name: Cannella Nera
SSR profile ID: 7

	OIV Code	Characteristic	Note	Description
Young shoot	001	Opening of the shoot tip	5	fully open
	004	Density of prostrate hairs on the shoot tip	5	medium
Shoot	016	Number of consecutive tendrils	1	2 or less
Young leaf	051	Color of upper side of blade (4th leaf)	3	bronze
Mature leaf	067	Shape of blade	4	circular
	068	Number of lobes	4	seven
	070	Area of anthocyanin coloration of main veins on upper side of blade	1	absent
	076	Shape of teeth	3	both sides convex
	079	Degree of opening/overlapping of petiole sinus	3-5	open - closed
	081-2	Petiole sinus base limited by vein	1	not limited
	084	Density of prostrate hairs between main veins on lower side of blade	7	high
	087	Density of erect hairs on main veins on lower side of blade	5	medium
Berry	223	Shape	2	globose
	225	Color of skin	6	blue black
Flower	151	Sexual organs	3	fully developed stamens and fully developed gynoecium

Sample name: Pizzutello Bianco.
True-to-type prime name: Zunek (Uva Sacra)
SSR profile ID: 8

	OIV Code	Characteristic	Note	Description
Young shoot	001	Opening of the shoot tip	5	fully open
	004	Density of prostrate hairs on the shoot tip	3	low
Shoot	016	Number of consecutive tendrils	1	2 or less
Young leaf	051	Color of upper side of blade (4th leaf)	3	bronze
Mature leaf	067	Shape of blade	2	wedge-shaped
	068	Number of lobes	2	three
	070	Area of anthocyanin coloration of main veins on upper side of blade	1	absent
	076	Shape of teeth	2	both sides straight
	079	Degree of opening/overlapping of petiole sinus	3	open
	081-2	Petiole sinus base limited by vein	1	not limited
	084	Density of prostrate hairs between main veins on lower side of blade	1	none or very low
	087	Density of erect hairs on main veins on lower side of blade	1	none or very low
Berry	223	Shape	3	broad ellipsoid
	225	Color of skin	1	green yellow
Flower	151	Sexual organs	3	fully developed stamens and fully developed gynoecium

Sample name: Montarale Vergari 1; Montarale Vergari 2.
True-to-type prime name: Lambrusca di Alessandria
SSR profile ID: 9

	OIV Code	Characteristic	Note	Description
Young shoot	001	Opening of the shoot tip	5	fully open
	004	Density of prostrate hairs on the shoot tip	5	medium
Shoot	016	Number of consecutive tendrils	1	2 or less
Young leaf	051	Color of upper side of blade (4th leaf)	1-3	green - bronze
Mature leaf	067	Shape of blade	4	circular
	068	Number of lobes	3	five
	070	Area of anthocyanin coloration of main veins on upper side of blade	3	up to the 1st bifurcation
	076	Shape of teeth	3	both sides convex
	079	Degree of opening/overlapping of petiole sinus	3	open
	081-2	Petiole sinus base limited by vein	1	not limited
	084	Density of prostrate hairs between main veins on lower side of blade	3	low
	087	Density of erect hairs on main veins on lower side of blade	5	medium
Berry	223	Shape	2	globose
	225	Color of skin	6	blue black
Flower	151	Sexual organs	3	fully developed stamens and fully developed gynoecium

Sample name: Palombaro Avi Amonzi.
True-to-type prime name: Malvasia Bianca Lunga
SSR profile ID: 11

	OIV Code	Characteristic	Note	Description
Young shoot	001	Opening of the shoot tip	5	fully open
	004	Density of prostrate hairs on the shoot tip	7	high
Shoot	016	Number of consecutive tendrils	1	2 or less
Young leaf	051	Color of upper side of blade (4th leaf)	1-2	green - yellow
Mature leaf	067	Shape of blade	2	wedge-shaped
	068	Number of lobes	3-4	five - seven
	070	Area of anthocyanin coloration of main veins on upper side of blade	1	absent
	076	Shape of teeth	3	both sides convex
	079	Degree of opening/overlapping of petiole sinus	7	overlapped
	081-2	Petiole sinus base limited by vein	1	not limited
	084	Density of prostrate hairs between main veins on lower side of blade	7	high
	087	Density of erect hairs on main veins on lower side of blade	1	none or very low
Berry	223	Shape	2	globose
	225	Color of skin	1	green yellow
Flower	151	Sexual organs	3	fully developed stamens and fully developed gynoecium

Sample name: Terracavata 25.
True-to-type prime name: Cargarello (Canaiolo Bianco or Drupeggio)
SSR profile ID: 16

	OIV Code	Characteristic	Note	Description
Young shoot	001	Opening of the shoot tip	5	fully open
	004	Density of prostrate hairs on the shoot tip	5	medium
Shoot	016	Number of consecutive tendrils	1	2 or less
Young leaf	051	Color of upper side of blade (4th leaf)	1-3	green - bronze
Mature leaf	067	Shape of blade	4	circular
	068	Number of lobes	3	five
	070	Area of anthocyanin coloration of main veins on upper side of blade	1	absent
	076	Shape of teeth	3	both sides convex
	079	Degree of opening/overlapping of petiole sinus	5	closed
	081-2	Petiole sinus base limited by vein	1	not limited
	084	Density of prostrate hairs between main veins on lower side of blade	7	high
	087	Density of erect hairs on main veins on lower side of blade	1	none or very low
Berry	223	Shape	2	globose
	225	Color of skin	1	green yellow
Flower	151	Sexual organs	3	fully developed stamens and fully developed gynoecium

Sample name: Casevecchie 3.
True-to-type prime name: Primitivo
SSR profile ID: 20

	OIV Code	Characteristic	Note	Description
Young shoot	001	Opening of the shoot tip	5	fully open
	004	Density of prostrate hairs on the shoot tip	7	high
Shoot	016	Number of consecutive tendrils	1	2 or less
Young leaf	051	Color of upper side of blade (4th leaf)	1	yellow
Mature leaf	067	Shape of blade	3	pentagonal
	068	Number of lobes	3-5	five - seven
	070	Area of anthocyanin coloration of main veins on upper side of blade	2	only at the petiolar point
	076	Shape of teeth	2	both sides straight
	079	Degree of opening/overlapping of petiole sinus	3	open
	081-2	Petiole sinus base limited by vein	1	not limited
	084	Density of prostrate hairs between main veins on lower side of blade	1	none or very low
	087	Density of erect hairs on main veins on lower side of blade	5	medium
Berry	223	Shape	2	globose
	225	Color of skin	6	blue black
Flower	151	Sexual organs	3	fully developed stamens and fully developed gynoecium
